# Etching: The Art of Semiconductor Micromachining

**DOI:** 10.3390/mi16020213

**Published:** 2025-02-13

**Authors:** Lucia Romano

**Affiliations:** 1 Institute for Biomedical Engineering, ETH Zürich, 8092 Zürich, Switzerland; lucia.romano@psi.ch; 2 Center for Photon Science, Paul Scherrer Institute, 5232 Villigen, Switzerland

Etching makes a pattern design a real 3D object. In semiconductor device fabrication, etching refers to any technology that selectively removes layers of material from a wafer’s surface, forming structures with specific depths and shapes. Etching is the process step that takes the design information from masks and transfers it into regions on the semiconductor where doping, isolation, gates, and metals are located. Depending on the complexity of the chip, a manufacturing work flow comprises several etching steps in dedicated tools distinguished by chemistries and processing capabilities. Etching plays a crucial role in various stages of semiconductor manufacturing, enabling to define features such as transistors, trenches for interconnects (e.g., IC) and wire bonding (e.g., packaging), microscale components with high precision and repeatability (e.g., MEMS).

Etching removes material through two basic mechanisms:

- Physically: ions with enough kinetic energy to sputter (or ion mill) atoms from the material;
- Chemically: etchant species form chemical reactions to promote the removal of atoms at the surface of the material.

Historically, wet chemical methods [1,2,3,4] were used to etch silicon until the advent of very-large-scale integration (VLSI) and ultra-large-scale integration (ULSI) technologies. VLSI and ULSI designs demand much more precise pattern transfer fidelity. In addition, aspect ratios (depth-to-width ratios) in advanced devices increased the ability to anisotropically etch material using directional etching technologies. Technically, dry-etching refers to the process of removing materials from the wafer surface using gaseous chemical etchants, which react with materials on the wafer to form volatile reaction products that are then evacuated from the reaction chamber. The etchants are usually generated directly or indirectly from the plasma of the etching gases. First proposed in the 1970s [5,6], reactive ion etching (RIE) uses etching gas species (e.g., SF6, CHF_3_, etc.) that are injected into a vacuum chamber and ionized (by radio frequency, microwave, etc.). The generated plasma contains ions and reactive radicals that are accelerated toward the substrate; this directional bombardment then favors anisotropic etching, transferring the pattern defined by lithography into the substrate. In plasma etching, multiple dynamic physical and chemical processes take place simultaneously and interact with each other, e.g., fluid dynamics of gaseous species, plasma generation and discharge, kinetic transport of ions and neutrals, chemical kinetics of etching reactions, adsorption and desorption of atoms and molecules on gas–solid interfaces, etc.

With the ongoing drive in the semiconductor industry towards miniaturization and more compact chips, the need for advanced etching techniques is more crucial than ever [7]. Etching equipment is a high-tech product created by integrating the most advanced technologies such as plasma generation, new materials, ultra-high vacuum, precision machining, automation, software control, etc. Plasma etching machines are among the most sophisticated tools right after lithographic tools [8].

Behind the huge investments in IC’s manufacturing, many other applications benefit silicon-etching methods, such as MEMS [9,10,11,12,13,14], microfluidics [15], micro-lenses [16], meta-surfaces [17], bio-interfaces [18], and X-ray optics [19,20,21,22]. The high demand for Si 3D microstructures with a high aspect ratio has driven both conventional (i.e., etching) and non-traditional [23] machining techniques to their maximum potential.

We refer to the most recent reviews about classical wet-etch [4] and dry-etch [24,25] methods for a comprehensive overview. Despite the fact that many other materials need to be etched for micromachining purposes—III-V compounds, many oxides, diamond, SiC, lithium niobate [26], tungsten [27], and aluminum [28]—the processing is less advanced than silicon-based manufacturing. New materials call for investigations into novel etch chemistry, quite often with the stringent necessity of integration on a silicon platform, as motivated by new advances in nanophotonics [29], quantum computing [30], and bio-chips [31,32]. As silicon is one of the most studied materials, its etching also represents the most used process of micromachining, spanning from high-aspect-ratio silicon nanostructures with non-Bosch processes and non-conventional wet-etching to several applications. The Bosch process is a high-aspect-ratio etching technology that repeats the cycle of isotropic etching followed by protection film deposition. The SF_6_ plasma cycle etches silicon, and the C_4_F_8_ plasma cycle creates a protection layer. An aspect ratio over 50, although it has been demonstrated, remains very challenging for the Bosch process. In pseudo-Bosch and non-Bosch processes, for example, DREM [33], the etching can achieve almost infinite selectivity, mainly due to the depletion of the C_4_F_8_ inhibitor, which is completely replaced in cryo-etching by oxygen [34], extending the micromachining capability of dry-etch to nanostructures [35]. The bottleneck of plasma etching is finally the isotropic nature of silicon’s reaction with fluorine species and the diffusion of the etchant species from the top of the pattern to the bottom of the structure with an increasing aspect ratio. Wet-etching [36], on the other hand, has the advantage of electrochemistry to directionally drive the reaction to create ordered macropore arrays [37,38] or elongated high-aspect-ratio silicon nanostructures [19,39,40]. In particular, metal-assisted chemical etching (MacEtch) [41,42,43,44] has recently been proposed as a new technology for structuring silicon at multiple scales [45] with many potential advantages with respect to plasma-based processing [46,47], such as incredible aspect ratio capabilities [48,49], low roughness [50], free of ion induced damage [51], suitable for III-V compounds [52], and a potentially dry [53] and CMOS-compatible [54,55] process. MacEtch has been successfully demonstrated in combination with electron beam [56,57], UV [19,44,58,59], flow-enabled self-assembly [60], tip-based [61], electrochemical nanoimprint [62], and chemisorption-assisted transfer printing [63], as well as displacement Talbot [64] lithography.

Depending on which specific etching technology, material process, and application demand are used, in general, there are still numerous challenges in the way of achieving highly precise and controlled etching. It is necessary to ensure that the whole etching process is both anisotropic and selective, compatible with the selected materials and application platform, and integrable with the whole process chain and environment. At the same time, it needs to be reproducible, cost-effective, and environmentally friendly for the benefit of our society and scientific progress.

## Abbreviations

The following abbreviations are used in this manuscript:

VLSI	very-large-scale integration
ULSI	ultra-large-scale integration
ICs	Integrated Circuits
RIE	reactive ion etching
DREM	Deposit Remove Etch Multistep
MEMSs	Micro-ElectroMechanical Systems
CMOS	Complementary Metal-Oxide Semiconductor
